# Production and characterization of volatile compounds and phytase from potentially probiotic yeasts isolated from traditional fermented cereal foods in Nigeria

**DOI:** 10.1186/s43141-020-00031-z

**Published:** 2020-06-08

**Authors:** Omotade Richard Ogunremi, Renu Agrawal, Abiodun Sanni

**Affiliations:** 1 Department of Biological Sciences, First Technical University, Ibadan, Nigeria; 2 Food Microbiology Department, Central Food Technological Research Institute, Mysore, India; 3 Department of Microbiology, University of Ibadan, Ibadan, Nigeria

**Keywords:** Volatile compounds, Phytase, Probiotics, Fermented food, Yeast

## Abstract

**Background:**

Probiotic strains are incorporated into food substrates to contribute to fermentation process. The technological suitability of such strains to improve the flavor and nutritional value of fermented food is strain-specific. Potentially probiotic yeasts isolated from Nigerian traditional fermented foods were assessed for production of volatile compounds by gas chromatography-mass spectrophotometry. Phytases were characterized for activity and stability at different pH (3–8) and temperatures (25-50 °C).

**Results:**

A total of 45 volatiles compounds were identified from intracellular cell-free extracts of *Pichia kluyveri* LKC17, *Issatchenkia orientalis* OSL11, *P. kudriavzevii* OG32, *P. kudriavzevii* ROM11, and *Candida tropicalis* BOM21. They include alcohols (14), carbonyls (13), esters (10), and organic acids (8). Phenylethyl alcohol was the highest higher-alcohol in *Issatchenkia orientalis* OSL11 (27.51 %). The largest proportion of esters was detected in *P. kudriavzevii* OG32 (17.38 %). *Pichia kudriavzevii* OG32 and *C. tropicalis* BOM21 showed vigorous gowth in minimal medium supplemented with sodium phytate (2 g L^−1^). Extracellular phytases from *P. kudriavzevii* OG32 and *Candida tropicalis* BOM2 showed optimal activiy at pH 4.6 (104.28 U) and pH 3.6 (81.43 U) respectively.

**Conclusions:**

Results obtained revealed species- and strain-specific potentials of the yeast strains to improve flavor and mineral bioavailability of fermented food products. Therefore, the application of these yeasts as starter cultures during food fermentation process is a very promising method to enhance the flavor profile and enhance mineral bioavailability in indigenous cereal-based fermented food products.

## Background

Probiotics are live microorganisms which, when administered in adequate amounts, confer health benefits on the host [1]. Indigenous fermented food products possess the potentials to meet the increasing demand for clean-label and health-beneficial foods by incorporating probiotic strains. Probiotics strains are typically selected from species of lactic acid bacteria and bifidobacteria [2]. However, the prospects of broader health benefits and delivery in unconventional food systems, including cereals, plant juices, and legumes have driven efforts toward exploring the larger microbial communities, including yeasts for microbial strains expressing interesting functionalities [3].

Several authors have reported the probiotic potentials of yeast strains from several indigenous fermented foods and beverages: *burukutu*, cheese, *fura*, *gowe*, *kunu*-*zaki*, *mawe*, *nunu*, kefir, *ogi*, olives, and wines [4–11]. Most of these yeasts are non-*Saccharomyces* species, including strains of *Debaryomyces hansenii*, *Issatchenkia orientalis*, *Galactomyces geotrichum*, *Kluyveromyces marxianus, K. lactis*, *Pichia farinosa*, *P. anomala*, *P. kudriavzevii*, and *Yarrowia lipolytica.* Their robust sizes (approx. 5 × 10 μm), stress tolerance, antibiotic resistance that is not due to mobile genetic materials, non-pathogenic, non-toxigenic and non-allergenic nature, versatile enzyme profile, and production of several bioactive compounds contribute to the selection of yeasts as probiotics.

Technological suitability, including contribution to appealing flavor and improvement of nutritional value, are desirable for the selection of probiotic strains to be incorporated in non-dairy food systems, especially cereal models [12]. Volatile organic compounds (VOCs) are flavor-active metabolic products of organic compounds in living cells, especially yeasts through specific pathways such as Ehrlich, β-oxidation, and glycolytic pathways [13, 14]. Recently, non-*Saccharomyces* yeasts are gaining attention as excellent producers of flavor compounds. They include some species of *Candida*, *Galactomyces*, *Geotrichum*, *Hanseniaspora*, *Pichia*, *Saccharomycopsis*, *Saprochaete, Starmera*, and *Wickerhamomyces* [13–15]. VOCs, including organic acids, esters, and higher alcohols determine the distinctive bouquet of the fermented product, contributing fruity, flowery, spicy, coffee to meaty nuance [14–18].

Phytate (Inositol hexaphosphate, IP6), the main form of phosphorous and a complex with dietary minerals (calcium, iron, magnesium, and zinc) in cereals, legumes, and nuts, is indigestible in the monogastric gastrointestinal tract [19, 20]. Phytate is considered to be the most important anti-nutritional factor for the bioavailability of dietary minerals to consumers with diets exclusively based on cereals [21]. It is implicated in the global burden of iron deficiency and the attending complications particularly among women and children in low-income countries. Bioavailability of dietary minerals may be improved by using phytase, an enzyme that catalyzes the sequential hydrolysis of phytate [22, 23].

Phytase-active probiotic strains have the potential to improve the in situ and in vivo bioavailability of the divalent minerals, during food fermentation and in the gastrointestinal tracts respectively [20, 24]. Several authors have reported high phytase production by yeasts from various food sources, including *Arxula adeninivorans*, *Hanseniaspora guilliermondii*, *I. orientalis*, *P. anomala*, *P. kudriavzevii*, *Saccharomyces cerevisiae*, and *S. pastorianus* [20, 25–28]. However, the application of yeast phytases is dependent on the stability of the enzyme to evolving conditions during food fermentation and gastrointestinal transit. Therefore, broad pH and thermal stability are important properties for the enzyme.

In a previous study, we determined the probiotic potentials of yeasts isolated from some cereal-based Nigerian traditional fermented food product [8]. These yeast strains were evaluated as platform strains for the production of volatile compounds and phytase. In addition, the activity and stability of extracellular phytases from selected strains were determined for possible application during food fermentation and gastrointestinal transit.

## Methods

### Reagents and materials

Solvents and other chemicals were of analytical grade. Sodium phytate was obtained from Sigma Chemical Co. (St. Louis, MO).

### Yeast strains and culture conditions

Yeast strains used in this study were selected based on the demonstration of probiotic potentials in a previous study [8]. They had been identified as *Pichia kluyveri* LKC17, *Issatchenkia orientalis* OSL11, *P. kudriavzevii* OG32, *P. kudriavzevii* ROM11, and *Candida tropicalis* BOM21 by sequencing D1/D2 region of large subunit of 26S rDNA gene. The GenBank accession numbers are KJ472904, KJ472906, KJ472905, KJ472907, and KJ472908 respectively [8]. Yeast strains were routinely grown on yeast peptone dextrose (YPD) media (per liter; 10 g yeast extract, 20 g peptone, 20 g glucose) (HiMedia, Mumbai, India) at 30 °C for 48 h.

### Fermentation conditions and preparation of pellets

A 1% (v/v) of 24-h-old broth culture of each yeast strain was inoculated in YPD broth and incubated at 30 °C till exponential phase (24 h). A 10 mL broth culture was centrifuged at 7500 rev min^−1^ and 4 °C for 10 min, the supernatant was discarded and yeast cells were washed twice with sterile saline water (0.85% NaCl).

### Analysis of volatile compounds

#### Extraction of volatile compounds from yeast cells

Extraction of volatile compounds was done by liquid-liquid extraction [29]. A suspension of yeast cells in 10 mL of dichloromethane was disrupted for 10 min with mortar and pestle at 5 °C and shaken vigorously in 100 mL separatory funnel for 2 min. The solvent phases were pooled into a dry test tube and dried with 0.5 g of anhydrous sodium sulfate. The extract was concentrated in a graduated tube to 500 μL by shaking in a water bath 25 °C.

#### Separation, identification, and quantification of volatile compounds

Volatile compounds in the concentrated extract were separated and detected by using a gas chromatography-mass spectrophotometer (GC-MS) (Perkin Elmer, Waltham, USA). The separation of volatiles was carried out in an ELITE 1 non-polar capillary column (30 m X 0.25 mm (ID); 0.25 μm film thickness). One microliter of extract was injected (split ratio 1:10) into the injection port and carried along the capillary column by helium gas (99.9%) at a flow rate of 1 mL min^−1^. The oven temperature was held at 100 °C for 6 min, heated at 4 °C min^−1^ to 150 °C, then at 8 °C min^−1^ to 220 °C and held at 220 °C until an approximate run time of 40 min.

The mass spectrophotometer was operated in the electron impact mode and mass spectra were taken using an ionization voltage of 70 eV. The mass scan range was 40–400 AMU, with a scanning speed of 0.2 s. Data acquisition and generation of chromatograms and mass spectra were done with the TurboMass software [29].

The identification of volatile compounds was performed by comparing the mass spectra with the standard spectra database from the NIST Ver. 2.1 2009 Mass Spectra Library. The proportion of each compound was calculated by comparing the peak area with the total area.

### Phytase analysis

#### Screening for phytase production

The test yeast strains were screened for phytase production by determining their ability to grow in a minimal medium with phytic acid as the sole source of phosphorus [27]. Yeast cells were harvested from 1 mL broth culture by centrifugation (7500 rev min^−1^ and 4 °C for 15 min) and cells were resuspended in 0.5 mL of sterile saline water. A 1% (v/v) of each cell suspension was inoculated into respective liquid growth media; phosphate-free minimal medium (per liter: 15 g glucose, 5 g NH_4_NO_3_, 2 g CaCl_2_, 0.5 g MgSO_4_.7H_2_O, 0.5 g KCl, 0.01 g FeSO_4_.7H_2_O, 0.01 g MnSO_4_.H_2_O) as negative control, phosphate-containing minimal medium (phosphate-free minimal medium + 3 g L^−1^ KH_2_PO_4_) as positive control and phytate-containing minimal medium (phosphate-free minimal medium + 3 g L^−1^ sodium phytate) as test. The broth cultures were incubated at 30 °C for 48 h. Yeast growth was determined after gentle agitation and measurement of the optical density at 600 nm [27]. This was carried out in triplicates and relative growth and was calculated using the formula below:

$$ \mathrm{Relative}\ \mathrm{growth}\ \left(\%\right)=\frac{Ai}{Ao}\times 100 $$

where *Ao* is the absorbance in phosphate-containing minimal medium and *Ai* is the absorbance in either phosphate-free or phytate-containing minimal medium.

#### Extracellular phytase extraction

A 1% (v/v) of an overnight culture of selected yeast strain was inoculated in a 250-mL Erlenmeyer flask, containing 100 ml of minimal salt medium (per liter: 15 g glucose, 5 g Na-phytate, 5 g NH_4_NO_3,_ 0.5 g MgSO_4_.7H_2_O, 2 g CaCl_2,_ 0.5 g KCl, 0.01 g FeSO_4_.7H_2_O, 0.01MnSO_4_.H_2_O). The medium was incubated in a shaking water bath (1700 rev min^−1^) at 30 °C for 48 h. Culture supernatant with extracellular phytase was obtained after the centrifugation (7500 rev min^−1^ and 4 °C for 10 min) of the broth culture and used for extracellular phytase assay [27].

#### Phytase activity assay at different pH

Phytase activity at different pH (3–8) was assayed by measuring the amount of inorganic phosphate liberated from sodium phytate in different buffer systems; 0.2 M citrate buffer (pH 3.0 and 6.0), 0.2 M acetate buffer (pH 3.5–5.5) and 0.2 M Tris–HCl buffer (pH 7.0–8.0). The reaction mixture consisted of 0.8 mL of the respective buffer containing 2 mM of Na-phytate and 0.2 mL of enzyme extract. Negative controls were prepared from enzyme extracts mixed with respective buffer without phytic acid. Reaction mixtures were incubated at 37 °C and stopped after 30 min by adding 1 mL of 10% trichloracetic acid (TCA). The blank was prepared by adding 10% TCA solution before the substrate was added. Determination of liberated inorganic phosphate was performed according to the ferrous sulfate-ammonium molybdate method [30]. Phosphate standard curve was prepared with inorganic phosphate (KH_2_PO_4_) (0-5 mmol mL^−1^). One unit (U) of phytase activity was defined as that which liberated one micromole of phosphate per minute under the assay conditions [26].

#### Phytase stability to different pH and temperature

Phytase was incubated at pH 3-8 for 1 h at 4 °C and different temperatures (20-50 °C) for 1 h. The residual phytase activity was assayed and relative activity was calculated [26].

## Results

### Volatile compounds produced by probiotic yeasts

Volatile organic compounds produced by test probiotic yeast strains are shown in Table 1. A total of 45 volatile compounds were identified and broadly categorized into four groups, including organic acids (8), alcohols (14), carbonyls (13) and esters (10). *Pichia kluyveri* LKC17 produced eighteen volatile compounds and carbonyls accounted for the highest number (7) and largest proportion (36%). The highest variety of alcohol was produced by *I. orientalis* OSL11. Phenyl ethyl alcohol was noted to be the largest proportion of alcohol produced by *I. orientalis* OSL11, *P. kudriavzevii* OG32, and *P. kudriavzevii* ROM 11. The largest proportion of esters was detected in *P. kudriavzevii* OG32 and it accounted for 17.38% of the total volatile compounds produced by the strain (Table 1).

**Table 1:** Analysis of volatile compounds produced by probiotic yeast strains

RT (min)	Compound name	Odor description^**a**^	Percentage (%) yield				
			***P. kluyveri*** LKC17	***I. orientalis*** OSL11	***P. kudriavzevii*** OG32	***P. kudriavzevii*** ROM 11	***C. tropicalis*** BOM21
	*Acids*						
4.72	Dimethyl-propanoic acid		1.79 ± 0.04	ND	ND	ND	ND
22.12	1,2-Benzenedicarboxylic acid		ND	0.30 ± 0.00	ND	ND	ND
22.83	Decanoic acid	Fatty	ND	ND	ND	ND	25.69 ± 3.01
25.86	9-Hexadecenoic acid	Fatty	ND	ND	5.66 ± 0.31	3.17 ± 0.00	ND
26.22	N-Hexadecanoic acid	Waxy fatty	20.38 ± 3.21	9.67 ± 0.11	10.60 ± 2.33	9.49 ± 0.45	3.88 ± 0.00
26.52	Octadecenoic acid	Fatty	0.80 ± 0.00	ND	ND	ND	ND
28.54	Erucic acid		12.50 ± 1.48	ND	ND	ND	ND
28.85	Nonadecanoic acid		ND	ND	ND	3.45 ± 0.00	ND
	**Total acids (8)**		**35.47 (4)**	**9.97 (2)**	**16.26 (2)**	**16.11 (3)**	**29.57 (2)**
	*Higher alcohols*						
3.37	1-Phenyl-1propanol	Floral, balsamic	ND	ND	ND	ND	0.40 ± 0.00
4.67	Phenyl ethyl alcohol	Floral, rosey	ND	27.51 ± 3.76	17.49 ± 1.04	17.38 ± 1.17	0.35 ± 0.00
18.99	DL-3,4-Dimethyl-3-4-hexanediol		0.67 ± 0.01	ND	ND	ND	ND
19.00	1-Decanol	Fatty, floral, orange	ND	0.39 ± 0.00	ND	ND	ND
21.30	2-Ethyl-1-decanol		ND	0.21 ± 0.00	0.49 ± 0.00	ND	2.37 ± 0.23
21.35	2-Hexyl-1-octanol		ND	ND	ND	ND	ND
21.88	4-Piperidine methanol		2.16 ± 0.42	1.02 ± 0.00	ND	ND	1.03 ± 0.00
22.56	1-Heptadecanol		ND	ND	ND	ND	8.29 ± 0.07
23.59	1-Dodecanol	Fatty, honey, coconut	ND	2.50 ± 0.01	ND	ND	ND
23.63	Cis-1,2-cyclohexanediol		1.22 ± 0.03	ND	2.52 ± 0.00	ND	ND
27.15	Nonacosanol		ND	ND	0.49 ± 0.00	ND	ND
27.17	1-Chloro-ethanol		ND	0.26 ± 0.00	ND	ND	ND
30.71	1-Pentacosanol		ND	0.69 ± 0.00	ND	ND	ND
31.42	(S)-3,4-Dimethylpentanol		0.32 ± 0.01	ND	ND	ND	ND
	**Total alcohols (14)**		**4.37 (4)**	**32.58 (7)**	**20.99 (4)**	**17.38 (1)**	**12.44 (5)**
	*Carbonyls*						
22.32	E-14-Hexadecenal		4.64 ± 0.06	0.31 ± 0.01	ND	ND	ND
23.13	Cis-oxacyclohexadecan-2-one		9.57 ± 0.71	ND	ND	ND	ND
23.15	Hexanal		ND	ND	ND	8.27 ± 0.59	ND
23.71	Heptanal		1.67 ± 0.00	ND	ND	1.50 ± 0.02	ND
24.97	Pyrrolo(1,2A)piperazine-1,4-dione		ND	11.64 ± 0.64	6.76 ± 0.21	9.52 ± 0.00	19.28 ± 2.00
25.00	1,2,5-Trimethyl-5-piperid-4-one		10.07 ± 0.89	ND	ND	ND	ND
25.11	Cyclohexanone		ND	6.12 ± 0.04	11.53 ± 4.00	ND	ND
25.17	3-Buten-2-one		5.63 ± 0.03	ND	ND	ND	ND
25.88	2-Heptadecenal		2.35 ± 0.10	5.25 ± 0.00	ND	ND	ND
28.45	E-11 Hexadecenal		ND	ND	ND	15.89 ± 4.66	ND
28.46	9-Octadecenal		ND	14.02 ± 1.00	16.63 ± 3.11	ND	ND
31.62	Decanal		ND	3.61 ± 0.07	ND	ND	ND
31.78	Dodecanal		2.07 ± 0.05	ND	ND	ND	ND
	**Total carbonyls (13)**		**36.0 (7)**	**40.95 (6)**	**34.92 (3)**	**35.18 (4)**	**19.28 (1)**
	*Esters*						
8.59	2-Phenylmethyl acetate	Honey, jasmine	3.86 ± 0.00	ND	ND	ND	ND
23.12	1-Methylphenyl butanoate	Jasmin, Apricot	ND	9.08 ± 0.84	10.17 ± 1.45	ND	ND
23.88	Tetramethyl acetate	Waxy, fruity, balsamic	ND	ND	ND	ND	2.18 ± 0.00
24.69	Isoamyl decanoate	Waxy, banana, fruity	ND	4.36 ± 0.20	4.51 ± 0.00	ND	7.89 ± 0.43
24.79	2-Bromo pentyl butanoate	Fruity	ND	ND	ND	3.54 ± 0.28	ND
25.12	2-Hydroxy,pentyl propanoate	Fruity, apricot, pineapple	ND	ND	ND	5.97 ± 0.11	ND
26.62	10-Undecen-1-yl hexanoate		0.40 ± 0.00	0.32 ± 0.00	ND	ND	ND
28.96	2-Ethyl octadecanoate	Waxy	4.84 ± 0.02	1.93 ± 0.00	2.70 ± 0.00	ND	ND
36.03	4-Heptenoic acid ethyl ester		ND	ND	ND	5.83 ± 0.11	ND
	**Total esters (10)**		**9.1 (3)**	**15.69 (4)**	**17.38 (3)**	**15.34 (3)**	**10.07 (2)**

*ND* not detected

^a^http://www.thegoodscentscompany.com/

### Screening for phytase production

**Fig. 1:**
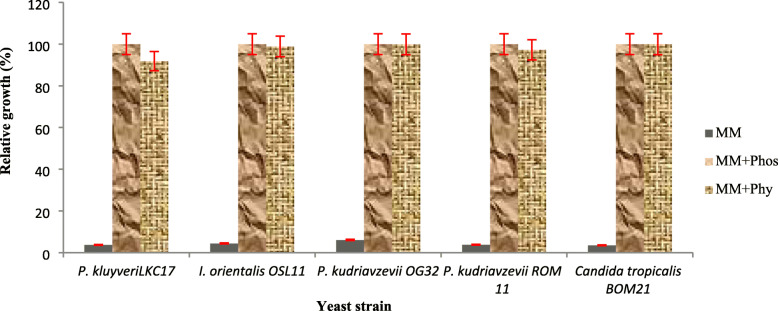
Relative growth of yeast at 30 °C after 48 h cultivation in MM, minimal salts medium; MM+ Phos, minimal salts medium supplemented with 3 g L^−1^ potassium dihydrogen phosphate (KH_2_PO_4_); MM+ Phy, minimal salts medium supplemented with 3 g L^−1^ sodium phytate

The test yeast strains were able to hydrolyze phytate in an enzyme-mediated reaction and utilized the generated myo-inositol phosphate intermediates (IP3–IP5) as sources of phosphorous for growth at 30 °C for 48 h. This was observed as increased cell density (600 nm) in respective broth cultures (Fig. 1). The relative growth of yeast strains in phytate supplemented minimal medium compared with phosphate supplemented minimal media was from the range of 91.82 to 99.93% while the relative growth in phosphate/phytate free minimal medium was less than 7% for all the yeast strains tested. *Pichia kudriavzevii* OG32 and *C. tropicalis* BOM21 had the higher relative growth of 99.88% and 99.93% respectively in phytate supplemented minimal medium and they were selected for phytase activity assay.

### Phytase activity and stability

**Fig. 2:**
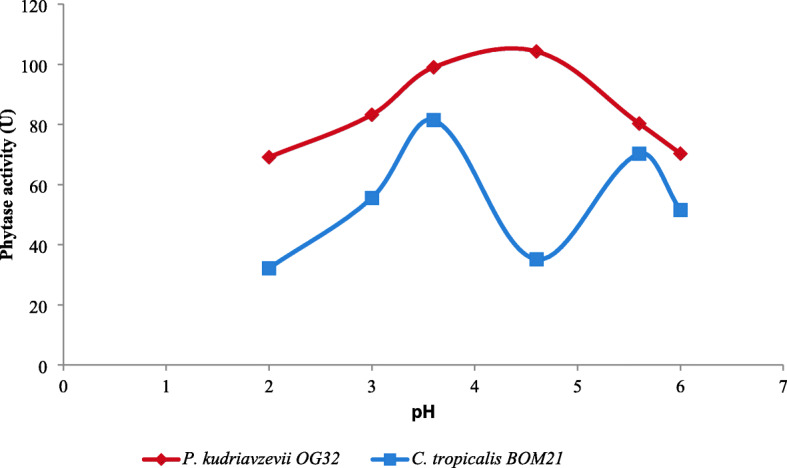
Extracellular phytase activity at different pH

**Fig. 3:**
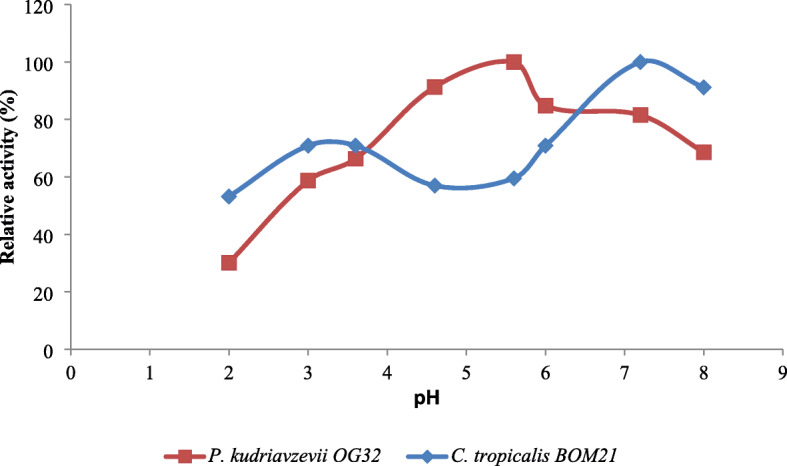
Extracellular phytase stability at different pH

The effects of pH based on three different buffer systems on extracellular phytase activity of both strains are shown in Fig. 2. The optimum activities were 81.43 U at pH 3.6 and 104.28 U at pH 4.6 for *C. tropicalis* BOM21 and *P. kudriavzevii* OG32 respectively. These indicate that they are acid phytases. Considering extracellular phytase from *P. kudriavzevii* OG3, a decline in activity was recorded as the pH move toward extreme acidic and neutral pHs. However, another peak (70.3 U) was recorded at pH 5.6 for *C. tropicalis* BOM21. Extracellular phytases from both test strains retained approximately 60% of optimal activity over a wide range of pH (3–8) (Fig. 3). The determination of the effect of prevailing temperatures during food processing (20, 30, and 50 °C) and in gastrointestinal tract (37 °C) on the stability of crude phytase extracts showed thermal stability (Table 2). Above 80% activity was retained at test temperatures.

**Table 2:** Stability of extracellular phytase from yeast strains at different temperature

Temperature (°C)	Relative phytase activity (%)	
	***P. kudriavzevii*** OG32	***C. tropicalis*** BOM21
20	79.25	100
30	87.42	97.14
37	100	87.14
50	90.57	91.43

## Discussion

In addition to the probiotic potentials of yeasts, some strains produce valuable metabolites that contribute to the sensory quality and nutritional value of fermented food products [31]. These are important criteria for selection as starter/adjunct cultures [32].

The potentially probiotic yeasts evaluated in this study showed strain-specific potentials to impart pleasant taste and distinct flavor on fermented foods by producing volatile organic compounds such as organic acids, alcohols, and esters. Previous studies reported species and strain specificity in the profile of flavor compounds produced by yeasts [33]. Among the organic acids, N-hexadecanoic acid was encountered in significant proportions in all the test yeast strains. This compound is widespread in yeasts [34]. It is characterized by a creamy fatty flavor and a dairy nuance [35]. In addition, N-hexadecanoic acid is listed as a flavor compound with GRAS status [36]. The production of phenyl ethyl alcohol was recorded in *I. orientalis* OSL11 and *P. kudriavzevii* OG32 and ROM11. It is one of the most abundant higher alcohols produced by yeasts, arising from the degradation of phenylalanine through the Ehrlich pathway [14, 18, 37]. Phenyl ethyl alcohol has a sweet floral taste and odor with rosey honey nuances [34]. Several of the higher alcohols identified in this study are listed as GRAS flavor compounds for food applications [36]. Esters are valuable compounds that impart characteristic fruity and flowery notes to fermented beverages [14, 16, 18]. In this study, *P. kudriavzevii* OG32 is the most dominant ester producer. Prominent among the esters from the strain is benzyl butanoate. It has a fruity aroma, specifically bringing about tropical, pineapple, and apple reminiscence [35]. In addition, methyl 2-phenyl acetate from *P. kluyveri* LKC17 and pentadecanoic acid 3 methylbutyl-acetate from *I. orientalis* OSL11, *P. kudriavzevii* OG32, and *C. tropicalis* BOM21 are important esters that were detected in significant proportions. Methyl 2-phenyl acetate is a high strength odor compound that is characterized by honey and jasmine aroma [35]. Similar to the observations from this study, high amounts of acetate esters were reported to be produced by strains of *P. kluyveri* and *P. kudriavzevii* from some other traditional fermented food sources [38, 39]. Other yeast species including *Starmera caribaea* and *Hanseniaspora guilliermondii* were reported to produce high levels of acetate esters with highly desirable flavor [13].

Phytase production has been previously detected and quantified with growth test in liquid medium supplemented with phytate salts as the sole source of phosphate [25, 27]. The higher relative growths of *P. kudriavzevii* OG32 and *C. tropicalis* BOM21 are in agreement with previous studies, being an indication of phytase production. *Pichia kudriavzevii* has been reported to produce cell-bound, intracellular and extracellular phytase [28, 40]. These species are predominant among the microflora associated with the spontaneous fermentation of several traditional fermented foods.

The maximum activity exhibited by phytases secreted by *C. tropicalis* BOM21 and *P. kudriavzevii* OG32 in the acidic pH range of pH 3.6-pH 4.6 are similar to the optimum pHs of phytases from other yeasts [26, 41, 42]. The technological suitability of the phytase-producing strains is dependent on the stability of secreted phytases to evolving pH and temperature during food fermentation and gastrointestinal transit. The significant portion of activity retained by phytases investigated over a wide range of pH (3–8) and temperatures supports the potentials of *C. tropicalis* BOM21 and *P. kudriavzevii* OG32 to remove phytate in diverse food fermentation models and the intestine.

## Conclusions

The volatile compounds identified to be produced by yeasts in this study are safe and possess relevant aroma for food use. In addition the phytase secreted by the yeast strains demonstrated activity and stability at conditions that prevail during food fermentation. Therefore, the application of these potentially probiotic yeasts as starter cultures during food fermentation process is a very promising method to enhance the flavor profile and enhance mineral bioavailability in indigenous cereal-based fermented food products.

## Data Availability

Not applicable.

## Abbreviations

GC-MS: Gass chromatography-mass spectrometer
ID: Internal diameter
IP6: Inositol hexaphosphate
NIST: National Institute of Standards and Technology
TCA: Trichloroacetic acid
VOC: Volatile organic compound
YPD: Yeast peptone dextrose
